# Long-Term Oncologic Outcome following Duodenum-Preserving Pancreatic Head Resection for Benign Tumors, Cystic Neoplasms, and Neuroendocrine Tumors: Systematic Review and Meta-analysis

**DOI:** 10.1245/s10434-024-15222-y

**Published:** 2024-04-05

**Authors:** Hans G. Beger, Benjamin Mayer, Bertram Poch

**Affiliations:** 1 c/o University Hospital Ulm, University of Ulm, Ulm, Germany; 2 Institute for Epidemiology and Medical Biometry, Ulm University, Ulm, Germany; 3 Centre for Oncologic, Endocrine and Minimal Invasive Surgery, Donau-Klinikum Neu-Ulm, Neu-Ulm, Germany

**Keywords:** Benign pancreatic head tumors, Cystic neoplasm, Neuroendocrine neoplasm of the pancreas, Periampullary neoplasms, Duodenum-preserving pancreatic head resection, Pancreatoduodenectomy

## Abstract

**Background:**

Pancreatoduodenectomy (PD) has a considerable surgical risk for complications and late metabolic morbidity. Parenchyma-sparing resection of benign tumors has the potential to cure patients associated with reduced procedure-related short- and long-term complications.

**Materials and Methods:**

Pubmed, Embase, and Cochrane libraries were searched for studies reporting surgery-related complications following PD and duodenum-preserving total (DPPHRt) or partial (DPPHRp) pancreatic head resection for benign tumors. A total of 38 cohort studies that included data from 1262 patients were analyzed. In total, 729 patients underwent DPPHR and 533 PD.

**Results:**

Concordance between preoperative diagnosis of benign tumors and final histopathology was 90.57% for DPPHR. Cystic and neuroendocrine neoplasms (PNETs) and periampullary tumors (PATs) were observed in 497, 89, and 31 patients, respectively. In total, 34 of 161 (21.1%) patients with intraepithelial papillar mucinous neoplasm exhibited severe dysplasia in the final histopathology. The meta-analysis, when comparing DPPHRt and PD, revealed in-hospital mortality of 1/362 (0.26%) and 8/547 (1.46%) patients, respectively [OR 0.48 (95% CI 0.15–1.58); *p* = 0.21], and frequency of reoperation of 3.26 % and 6.75%, respectively [OR 0.52 (95% CI 0.28–0.96); *p* = 0.04]. After a follow-up of 45.8 ± 26.6 months, 14/340 patients with intraductal papillary mucinous neoplasms/mucinous cystic neoplasms (IPMN/MCN, 4.11%) and 2/89 patients with PNET (2.24%) exhibited tumor recurrence. Local recurrence at the resection margin and reoccurrence of tumor growth in the remnant pancreas was comparable after DPPHR or PD [OR 0.94 (95% CI 0.178–5.34); *p* = 0.96].

**Conclusions:**

DPPHR for benign, premalignant neoplasms provides a cure for patients with low risk of tumor recurrence and significantly fewer early surgery-related complications compared with PD. DPPHR has the potential to replace PD for benign, premalignant cystic and neuroendocrine neoplasms.

**Supplementary Information:**

The online version contains supplementary material available at 10.1245/s10434-024-15222-y.

Pancreatic cystic neoplasms represent an increasingly detected entity of tumors of 10–15% of all pancreatic cystic lesions.^1^ Intraductal papillary mucinous neoplasms (IPMNs), mucinous cystic neoplasms (MCNs), and solid pseudopapillary neoplasms (SPNs) are primarily benign tumors but have variable inherent risks of malignancy. Serous cystic neoplasms (SCNs) are considered to be of benign nature. The risk for malignant transformation ranges up to 60%^2^ for main-duct (MD) or mixed-type IPMN, 6–46%^3^ for branch-duct (BD) IPMN, and up to 15%^4^ for MCN. IPMNs are predominantly located in the pancreatic head, whereas MCNs are detected more frequently in the body and tail. SPN are classified as low-grade malignant neoplasms, although 12–18% are malignant tumors in the final histopathology after surgical treatment.^5^

Pancreatic neuroendocrine neoplasms (PNETs) account for approximately 2% of all pancreatic tumors.^6^ They are heterogeneous neoplasms with a variable malignant potential for non-functional and functional neoplasms. Approximately 30–40% of pancreatic neuroendocrine neoplasms are located in the pancreatic head and neck.^7^ Goals of surgical treatment of benign and premalignant cystic neoplasms are cure of the patient, relief from symptoms by resection, prevention of development of a malignant pancreatic tumor, low risk for surgery-associated complications, and maintenance of pancreatic and upper-gastrointestinal tract (GI tract) tissues and functions.

The high level of surgical techniques and standardization, high quality of intensive care unit management, use of nonoperative interventions for complications, and surgical expertise in many centers have led to the consideration of Whipple resection or pylorus-preserving pancreaticoduodenectomy (PPPD) with increasing acceptance as the appropriate surgical treatment for benign tumors and premalignant cystic neoplasms (CNs) of the pancreatic head.^8,9^ However, the use of PD for patients suffering benign, premalignant, cystic, or neuroendocrine neoplasms questions the classical surgical treatment with respect to multiorgan tissue loss for a benign, local pancreatic disease.^10^ Despite a decrease in reported mortality and local complications, PD remains a complex, multiorgan resection with distinct surgery-associated complications, considerable mortality, and late metabolic morbidity.^10–16^ The development and increasing use of parenchyma-sparing, local resection of benign pancreatic tumors—tumor enucleation (TE),^17^ duodenum-preserving pancreatic head resection (DPPHR)^18^ and pancreatic middle segment resection^19^—parallels the increase in the number of patients with symptomatic or accidentally detected, asymptomatic, benign neoplasms requiring surgical treatment. The rationale for local resection of benign tumors of the pancreatic head are (i) cure of patients; (ii) preservation of the duodenum, extrahepatic biliary ducts, gastric antrum, and pylorus; (iii) maintenance of the functional integrity of the duodenum regarding coordination of digestive metabolic and motility functions; and (iv) maximum conservation of the pancreatic tissue. DPPHR for benign pancreatic head tumors has the advantage of conservation of the duodenum and first jejunal loop and a limited loss of pancreatic and biliary tissues. New onset of diabetes mellitus (DM) and new onset of pancreatic exocrine insufficiency (PEI) are assessed to be low following DPPHR;^18^ in most patients, endocrine and exocrine functions were measured as being at the preoperative level.^20–22^ Many institutions reported low rates of surgery-associated complications regarding frequencies of reoperation, reintervention, and in-hospital mortality after DPPHR.^20^ Maintenance of endocrine and exocrine pancreatic and upper-GI tract functions are documented by studies with high clinical evidence.^21,22^ However, data regarding oncologic outcome following DPPHR are lacking. Consequently, this systematic review and meta-analysis aims to evaluate the pattern of long-term outcomes regarding tumor recurrence and late mortality comparing DPPHR and PD. The hypothesis was that DPPHR applied for benign tumors ensures the cure of patients, and is, compared with data following PD, associated with a low risk for procedure-related surgical morbidity. The primary endpoints were the metrics for postoperative complications and for oncologic outcome, frequency and type of tumor recurrence, anastomotic tumor recurrence, and tumor reoccurrence in the remnant pancreas based on the final histopathology after DPPHR or PD.

## Materials and Methods

### Search Strategy

We conducted a comprehensive literature search of the PubMed/Medline, Embase, and Cochrane databases. For PubMed, a search for medical subject heading (MeSH) terms was applied. For Embase and Cochrane, searches with Emtree and MeSH terms were performed, respectively, including a text word search for surgical techniques. A text word search for pancreatic resection techniques including duodenum-sparing head resection and pancreatoduodenectomy for benign tumors was performed. The following search items were used: duodenum-preserving pancreatic head resection, parenchyma-sparing surgery for pancreatic head tumors, pancreatoduodenectomy for benign tumors, Whipple resection for cystic neoplasms, Whipple resection for neuroendocrine tumors, pancreatic head resection with segment resection of the duodenum, local resection of periampullary tumors, severe dysplasia, and advanced cancer in resected benign pancreatic head tumors.

**Fig. 1:**
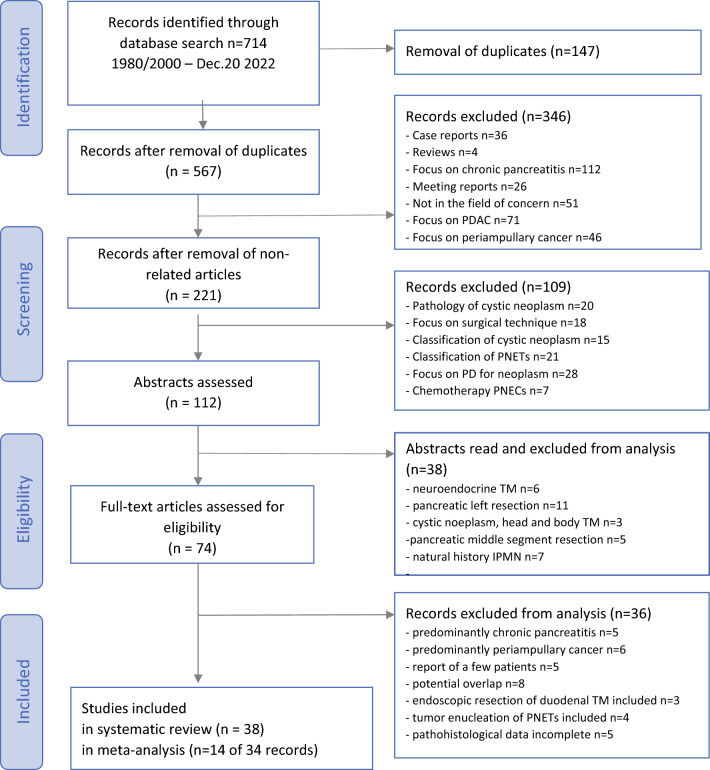
PRISMA flow diagram on the selection process of studies

Studies reporting limited surgery for cystic neoplasms, neuroendocrine tumors of the pancreatic head, or low-risk periampullary tumors were included in the selection process. The preoperative diagnosis and final histological diagnostic pattern of benign tumors of the pancreatic head included IPMN, MCN, SPN, serous cystic adenoma (SCA), non-functional and functional PNETs, periampullary tumors, inflammatory tumors of chronic pancreatitis, and other tumors. The search results for identification of relevant publications are presented in Fig. 1. Case reports, case series up to four patients, reports of assessment of metabolic functions after pancreatic head resection, and studies including advanced pancreatic head tumors in preoperative diagnosis were excluded. Figure 1 shows the PRISMA flow diagram of the selection process.^23^ The publications were checked for cross references that were eligible as additional reports that were not identified by the primary search items. Differences were resolved by mutual agreement between two authors (H.G.B. and B.P.).

### Evaluation of Methodological Quality of Studies

The methodological quality of the 38 studies finally included in the systematic review and of the 14 studies included in the meta-analysis was assessed using the Critical Appraisal Skills Program of the Oxford Centre for Evidence-Based Medicine.^24^ The manuscripts were evaluated according to this program for the level of evidence; specifically, criteria for selection bias, measure bias, and applicability were assessed for each study. Additionally, the Newcastle-Ottawa Scale (NOS) was applied to assess the quality of the controlled, prospective, and retrospective cohort studies, ensuring an objective evaluation of the most basic quality aspects of non-randomized cohort studies with regard to selection criteria, case definition, representativeness of cases, application of international histopathological criteria, selection of controls, comparability of study groups, and assessment of outcome variables.^25^ Cohort studies with scores of 8 or 9 were considered to have good-to-high levels of evidence and were included in the analysis (Tables 1 and 2).

**Table 1:** Baseline data and quality assessment of the review group DPPHR for benign tumors, cystic neoplasms, neuroendocrine tumors, and papillary/ampullary tumors of the pancreatic head

Reference no.	Publ. year	Study period	DPPHR total pats *N*	Age years (mean)	M/F	DPPHR total/partial	Type of cohort study	Quality assessment	
								Oxford evidence	NOS
Chao Lu [28]	2022	2015–2021	25	46.7 ± 17.7	8/17	Partial 24^†^ Total 1	Prospective	3	8
Min Zhou [29]	2022	4–12/2020	30	39.4	14/16	Total^†^	Prospective	2b	8
Hong Deifei [30]	2021	2016–2019	22	46.7 ± 16	6/16	Total 17^†^ Partial 5	Prospective	2c	8
Xuan Lin [31]	2021	2018–2020	5	45.2	1/4	Total^†^	Prospective	3	7
Cai Y [32]	2021	2–11/2019	24	43	9/15	Total^†^	Prospective	2b	9
Cao J [33]	2019	2016–2017	12	37.3	2/10	Total^†^	Prospective	2c	8
Snajdauf [34]	2019	1994–2015	13	14.9	3/10	Partial	Prospective	3a	8
Milanetto [35]	2016	1991–2015	8	33	5/3	Partial	Prospective	3b	8
Thomas [36]	2015	2008–2013	5	64	ND*	Partial^†^	Prospective controlled ^††^	2c	8
Kozlov [37]	2014	ND	16	ND	ND	Partial^†^	Retrospective^††^ controlled	3a	7
Suzuki [38]	2013	200–2012	5	54.5	1/5	Total	Retrospective	3a	7
Tsuchikawa [39]	2013	1994–2011	21	61	8/13	Total	Prospective	2c	8
Nakaghori**[40]	2010	1994–2007	15	64	13/2	Partial	Retrospective	3a	7
Beger [41]	2008	1982–2006	15	44	6/9	Total 11 Partial 4	Prospective	2c	8
Xiong [42]	2007	2001–2006	22	49.7	9/13	Partial	Prospective	2c	8
Fernandez-Cruz [43]	2006	1995–2006	8	65	ND	Total 4 Partial 4	Prospective^††^ controlled	2c	8
Ito [44]	2005	1998–2001	10	ND	8/2	Total	Retrospective^††^ controlled	2c	8
Murakami [45]	2004	ND	8	63 ± 13	7/1	Total	Retrospective	3b	7
Hirano [46]	2004	1989–1998	13	59	9/4	Total	Retrospective	3c	7
Takada [47]	2004	1988–2002	26	53.3/59.1	15/11	Total	Prospective	2c	7
Isaji [48]	2001	1996–1999	18	ND	ND	Total	Retrospective	3a	7
Naritomi [49]	1996	ND	6	57	4/2	Total	Prospective ^††^ controlled	2c	8
Imaizumi [50]	1995	1989-1993	20	52.5	10/10	Total	Prospective^††^ controlled	2a	9
Harada [51]	1994	1989–1991	15	ND	ND	Total	Prospective^††^ controlled	2c	8

*ND* no data

**Partial head resection processus uncinatus

^†^ Laparoscopic DPPHR

^††^Control group: [36], 12 pats. enucleation; [37], 15 pats. DPPHRt+SD; [43], 13 pats. Whipple; [44], 10 pats. Whipple; [49], 9 pats. PPPD; [50], 19 pats. PPPD; [51], 14 pats. PPPD

**Table 2:** Baseline data and quality assessment of studies included in meta-analysis: comparison of PD and DPPHR for benign tumors, cystic neoplasms, neuroendocrine tumors, and papillary/ampullary tumors of the pancreatic head

Author	Publication year	Study period	Total patients	Age	Gender	PD		DPPHR		Type of cohort study	Quality assessment	
						Patients	Type	Patients	Type		Oxfordevidence	NOS
			*N*	Years(mean)	M/F	*n*		*n*				
Wei Liu^52^	2022	2014–2022	50	58.5 ± 10	36/14	38	Whipple/PPPD	12	Total †	Retrospective controlled	3a	8
Sun Jonhui^53^	2020	2014–2018	86	45 ± 13	43/43	57	Whipple	29	Partial	Prospective controlled	2a	9
Chen^54^	2020	2016–2019	54	54.7 ± 13.9	28/26	39	Whipple	15	Total†	Retrospective controlled	2c	8
Qin^55^	2020	2007–2018	28	10.8–12	ND	6	PPPD	22	Total	Retrospective controlled	2c	8
Jiang Yu^56^	2018	2016–2016	68	47 ± 14.7	21/47	34	PPPD	34	Total †	Prospective controlled	2b	9
Li Yatong^57^	2017	2008–2014	62	49.5/49.8	29/33	42	PPPD	20	Total	Prospective controlled	2b	8
Perinel*^58^	2014	2007–2012	39	59/64	17/22	24	PPPD	15	Total 13 Partial 2	Retrospective controlled	3a	9
Liu^59^	2013	ND	57	49.3/46.2	34/23	31	PPPD	26	Partial	Retrospective controlled	2c	9
Gong^60^	2013	1998–2011	58	40 ± 12 45 ± 10	22/36	40	PPPD	18	Partial	Retrospective controlled	2c	8
Pedrazzoli^61^	2011	1991–2008	64	54 61	33/31	37	PPPD	27	Total 23 Partial 4	Prospective controlled	2a	9
Fujii^20^	2011	1991–2009	132	51/62.4	80/52	55	PPPD	77	Total	Prospective controlled	2a	9
Busquets**^62^	2010	1989–2006	62	51/46	42/20	41	Whipple 17 PPPD 24	21	Partial	Prospective controlled	2b	9
Horiguchi^63^	2010	ND	40	67/59	22/18	19	PPPD	21	Total	Prospective controlled	2b	9
Lee^64^	2010	1995–2007	100	56/47	42/58	70	PPPD	30	Total 16 Partial 14	Prospective controlled	2a	9

*In 9 and 5 patients who underwent DPPHR or PPPD, respectively, total pancreatectomy was done.

**Three patients with TM enucleation included.

^†^Laparoscopic or robotic-assisted DPPHR.

### Duodenum-Preserving, Total, or Partial Pancreatic Head Resection

DPPHRt involves resection of the pancreatic head while conserving the pancreatic neck, intrapancreatic common bile duct (CBD), and duodenum (Supplemental Material, Fig. S2A). A subgroup of DPPHRt comprises patients who underwent resection of the peripapillary segment of the duodenum (DPPHRt+sd) and resection of the intrapancreatic CBD (Supplemental Material, Fig. S2B). A few patients who underwent near total pancreatic head resection by conserving some suprapapillary pancreatic tissue of the groove of the pancreas are included in the DPPHRt group. Partial pancreatic head resection (DPPHRp) was performed when tumor size and the proposed biological nature of the neoplasm necessitated tissue resection extending beyond the pancreatic main duct. DPPHRp does not require resection of the duodenum and/or CBD; the tissue outside of the tumor wall of the ventral or dorsal pancreatic head is preserved (Supplemental Material, Fig. S2C). Reconstruction techniques were predominantly pancreatico-jejunostomy, pancreatico-gastrostomy, and pancreatico-duodenostomy.

### Data Extraction Process

The presented data are based on a selective evaluation of 38 studies dealing with DPPHR published between 1994 and 2022. Data extraction from each study was conducted independently by two authors (H.G.B. and B.P.) according to the lists of prespecified selection criteria. To evaluate the intraoperative and early postoperative outcomes, the following criteria were used for analysis: in-hospital mortality, reoperation, and tumor size.

The final histology of the tumors was listed separately, including IPMN, MCN, SPN, SCA, and pancreatic non-functional and functional PNETs, as well as periampullary tumors originating from the peripapillary duodenum, papilla or ampulla, and prepapillary CBD. Chronic pancreatitis and other cysts and tumors were additionally listed. Advanced pancreatic cancer, preoperatively considered to be benign tumor, but identified by frozen section investigation intraoperatively and/or by final histopathological diagnosis, was listed separately in the respective group of neoplasm: IPMN, MCN, and PNET and PAT. For IPMN and MCN, specific criteria with respect to MD- or BD-IPMN and the degrees of dysplasia and minimal invasive or focal carcinoma were listed separately and included in the analysis when clear definitions were reported in the manuscript. High-grade dysplasia of IPMN and MCN were listed as benign, avoiding the term carcinoma in situ.^26^ Regardless of the staging of the reports (minimally invasive, focal, or micro-carcinoma or T1–2 carcinoma), we used the term “cancer arising in association with IPMN” (“IPMC”) or “invasive cancer” for cancer. With respect to long-term outcome after DPPHR or PD for benign tumors, the type and frequency of tumor recurrence were listed. Specifically, a recurrent tumor at the resection margin, metachronous reoccurrence of tumor in the remnant pancreas, and extrapancreatic metastazation were documented separately as tumor recurrence. All patients with advanced cancer identified by frozen section or/and final histopathologic assessment experienced either a conversion to PD intraoperatively or early postoperative re-surgery by PD and/or DPPHR plus adjuvant chemotherapy during the index hospitalization; these patients were kept in the follow-up registry of the cohorts.

The indication for DPPHR or PD was based on the presence of abdominal symptoms in approximately 85% of patients. All tumors were considered preoperatively to be of benign nature, except some patients with papillary/ampullary tumors (PATs). Periampullary tumors were subdivided into tumors derived from the peripapillary duodenum, adenomas of the papilla/ampulla, or tumors of the peripapillary CBD, including pancreaticobiliary maljunction. Evaluating long-term outcomes, data regarding time, and reason for late mortality during the reported follow-up period were separately listed. Seven authors were contacted to clarify the cause and type of postoperative interventions and histological classification of the tumor that were lacking in their respective publications.^43,45,55,56,58,60,63^ The reported period covers 27 years. The criteria grade of dysplasia, BD-, MD-, and mixed-type IPMN, and type of recurrent tumor are incompletely reported because the clinical histopathological criteria were only established as guideline metrics in recent years.

### Statistical Analysis

All analyses were conducted using R for statistical computing version 4.2.2 (www.r-project.org, package meta). Continuous variables were expressed as mean ± standard deviation (SD), whereas categorical variables were presented as absolute frequencies and percentages. Explorative statistical testing of the DPPHR subgroups (total versus partial resection) was performed using the chi-squared test. Statistical significance was set at *p* < 0.05. For the meta-analytic approach, odds ratio (OR, Mantel–Haenszel method) was used for all considered dichotomous outcomes.^27^ All effect estimates were presented together with their 95% confidence intervals (CIs). To assess the extent of between-study heterogeneity, the *I*^2^ statistic was evaluated, leading to the application of a fixed-effects model where *I*^2^ was < 40%; otherwise, a random effects model was used. A graphical representation of the results was based on forest plots. To determine whether significant publication bias had to be assumed, funnel plots were additionally created.

## Results

### Study Groups

The analysis was based on 38 good- to high-quality cohort studies, presenting data from 729 patients following DPPHR (Tables 1 and 2). A total of 533 patients included in the meta-analysis underwent PD for benign tumors, premalignant neoplasms, or low-risk malignant periampullary tumors. The systematic review was performed by analyzing the DPPHR-related data of all patients of the 38 cohort studies. DPPHRt was performed in 499 patients and DPPHRp in 230 patients. The meta-analysis was based on data from 14 controlled studies, including a control group of patients who underwent PD. In the meta-analysis, the results of 367 patients following DPPHR were compared with 533 patients following PD, of whom 151 underwent Whipple resection and 382 PPPD.

### Assessment of Methodological Quality of Studies

The systematic review was based on 24 cohort studies in the review group (Table 1) and on 14 studies in the meta-analysis group (Table 2). In total, 21 studies were controlled cohort studies, of which 14 were prospective and seven retrospective reports. Seventeen reports were without a control group, of which 12 were prospective studies. The critical appraisal for methodology revealed 26 studies with evidence level 2 and 12 studies with evidence level 3. Evidence level 2 certifies a good-quality cohort study. Additionally, NOS score was applied to assess the quality of all cohort studies, which enabled an objective evaluation of the most basic quality aspects of nonrandomized studies. In total, 30 cohort studies elicited a score of ≥ 8; mean NOS score was 8.1, which indicated a good quality of the cohort studies.

### Results of Baseline Data

The baseline data of the 38 cohort studies, composed of 1262 patients, are presented in Tables 1 and 2. These studies included data from 729 patients following DPPHR and 533 patients following PD for benign tumors. In total, 27 studies were published between 2010 and 2022. The concordance of preoperative diagnosis of benign tumors and the final histopathology after DPPHR was 90.57%. Patients who experienced DPPHRp for chronic pancreatitis were excluded from the calculation of concordance. In the review group (Table 1), the mean age of the patients was 49.7 (SD ± 12.6) years and in the meta-analysis group, the mean age was 49.9 (SD ± 12.5) years (Table 2). The gender relationship M:F was 1:1.75 in the review group and 1:1.04 in the meta-analysis group. Two studies reported results after the application of DPPHR in adolescents and children, predominantly for SPN.^38,57^

### Results of Tailored Use of Duodenum-Preserving Pancreatic Head Resection

In total, 499 patients (68.4%) underwent DPPHRt and 230 (31.6%) DPPHRp (Table 3). Tumor size of the DPPHRt group was significantly larger than in the DPPHRp group (3.7 cm versus 2.9 cm, respectively, *p* = 0.001). In the DPPHRt group, 367 patients experienced complete preservation of the duodenum, and 173 patients underwent resection of the peripapillary segment of the duodenum and the CBD (DPPHRt+sd). In 189 patients who underwent DPPHRp, the duodenum and the intrapancreatic CBD were preserved, except in 7 patients, who underwent additional CBD resection.

**Table 3:** Tumor type based on histopathology of 729 patients following DPPHR for benign and premalignant neoplasms

	Patients	Cystic neoplasms					PNETs Functional + non-functional Benign/malignant	Periamp. tumors papilla/ampulla duodenum CBD Benign/malignant	Other tumors		TM size	Follow-up time
		Benign				Malignant**						
	*N*	IPMN *n*	MCN *n*	SPN *n*	SCN *n*	IPMC/MCC/ PDAC	*n*	*n*	Chronic pancreatitis*n*	Other TMs*** *n*	cm mean	Months mean
Total DPPHR*	499	196	39	45	52	22	55/7	13/12	47	11	3.7	38.4 ± 23.4
Partial DPPHR	230	62	18	30	30	3	23/4	6/0	48	6	2.9	46.0 ± 33.3

*DPPHRt/DPPHRt+sd: duodenum-preserving total head resection; DPPHRt+sd: segment resection of peripapillary duodenum.

**IPMC cancer arising in association with IPMN, *MCC* cancer in association with MCN, *PDAC* pancreatic ductal adenocarcinoma

***Other TMs includes metastases of renal cell carcinoma two pats.; ileum carcinoid metastasis one pat.

In-hospital mortality was 0% after DPPHRp. Following DPPHRt, 1/382 (0.26%) patients died (Supplemental Material, Fig. S2A). The frequency of reoperation was significantly lower following DPPHRt, with 3.3% versus 6.8% following PD [OR 0.52 (95% CI 0.26–1.04); *p* = 0.04].

GI tract reconstruction was performed with end-to-end anastomosis of the duodenum in 173 patients; in 199 patients an anastomosis of the CBD with the duodenum was performed. The GI tract reconstruction with the left pancreas was carried out with an excluded jejunal loop in 441 patients, with the stomach in 206 patients, with the duodenum in 67 patients, and as a duct-to-duct anastomosis of the pancreatic main duct (PMD) in 15 patients. In ten patients, duodenum-preserving total pancreatectomy was carried out with conservation of the spleen.

Of the 533 patients who underwent PD for benign tumors of the pancreatic head, Whipple resection was performed in 132 and PPPD in 401 (Table 2). When undergoing PD for benign tumors of the pancreatic head, in most patients pancreaticojejunostomosis was performed, in 104 patients a pancreaticogastrostomosis was performed, and in 181 patients a laparoscopic or robotic-assisted DPPHR was performed.

The final histopathologic diagnosis revealed 472 patients with benign CNs. Of these patients, advanced cancer was revealed in 25, including IPMN-associated minimally invasive, micro-, or focal cancer in 9; T_1/2_ in 14; and MCC and PDAC in 1 each. A total of 78 patients displayed a benign PNET in the final histopathology, of whom 55 presented a non-functional, benign neoplasm. A total of 11 patients from the PNET group displayed a malignant tumor: malignant gastrinoma 1 patient, carcinoid of the papilla in 4 patients, carcinoid of the duodenum in 1 patient, and islet cell carcinoma in 5 patients. A total of 31 patients displayed tumors of the papilla/ampulla or peripapillary CBD and/or maljunction of the pancreatic and biliary ducts. Other tumors were reported for 112 patients (Table 3), with chronic pancreatitis in 95 of these patients. Under ‘other tumors', patients were operated on with the diagnosis of benign neoplasm, and three patients presented histopathologically a metastasis of renal cell carcinoma (two patients) or metastasis of an ileum carcinoid (one patient). Of 18 patients who displayed advanced cancer intraoperatively or by final histological assessment of the operative specimen, 6 patients had a conversion to classical Whipple OP, 2 patients had re-surgery PD during the index hospitalization, and 10 patients experienced an additional adjuvant chemotherapy period.

### Results of Histopathological Assessment of the Operative Specimen following DPPHR

In total, 258 patients revealed IPMN and 57 patients MCN (Table 3). The IPMN differentiation in MD- and BD-duct and mixed-type IPMN was reported for 137 patients. Of them, 111 underwent surgery for BD-IPMN (81.0%). Predominantly DPPHRt or DPPHRt plus segment resection of the peripapillary duodenum was performed for MD- or BD-IPMN. With respect to the degree of dysplasia, 34 of 161 patients with IPMN (21.1%) were assessed to have severe dysplasia, whereas for MCN only 1 of 57 patients displayed severe dysplasia (Table 4). Advanced cancer in IPMN or MCN was found in 25 patients. In 13 of the 25 patients who exhibited advanced cancer intraoperatively by frozen section or by final histopathologic assessment of the operative specimen, 6 patients underwent a conversion to classical Whipple procedure and 2 patients underwent re-surgery during the index hospitalization applying a radical PD. Five patients received additional adjuvant chemotherapy after total DPPHR.

**Table 4:** Final histopathology and oncologic long-term outcome of 340 patients following DPPHR for IPMN and MCN of the pancreatic head—tumor recurrence after follow-up 45.8 months (mean)

		IPMN						IPMC*	MCN				MCC**	Recurrence
	IPMN Benign	MD	BD	Mild/ moderate dysplasia		Severe Dysplasia		Pats.	Pats. Benign	Mild/moderate dysplasia		Severe dysplasia		
	Pats.	Pats	Pats	Pats.		Pats.				Pats.		Pats.	Pats.	Pats.
	*N*	*n*_1_	*n*_1_	*n*_2_		*n*_2_		*n*	*N*	*n*		*n*	*n*	*n*_3_
DPPHRt/ DPPHRt+sd	196	23	78		127/161		34/161	21*	39		56/57	1	0	9/257 3.5%^a^
DPPHRp	62	3	33					3*	18			0	1	5/83 6.0%^b^

n_1_: IPMN MD-BD differentiation reported: ref. no. 56, 57, 47, 44, 48, 39, 64, 63, 61, 51, 30, 29, 28, 32, 62, 53

n_2_: IPMN grade of dysplasia not reported: ref. no. 28, 36, 53, 61, 44, 63, 51

n_3_: Recurrence of IPMN and MCN not reported: ref. No. 42, 37, 32, 54, 44, 47, 49

^a^: 1 pat. IPMN anastomosis, 2 pats. IPMC anastomosis, 3 pats. IPMN remnant pancreas, 2 pats. extrapancreatic metastasis, 1 pat. PDAC remnant pancreas

^b^: 2 pats. IPMN remnant pancreas, 1 pats. MCC anastomosis, 1 pat. IPMC remnant pancreas, 1 pat. peritoneal metastasis of IPMC

^*^IPMC: cancer arising in association with IPMN minimal invasive/focal carcinoma 9 pats.; IPMC T_1/2_ 14 pats.

PDAC T_2_ 1 pat.

^**^MCC: T_2_-cancer in association with MCN; 1 pat.

The final histopathology of 89 patients with PNETs following DPPHR is presented in Table 5. In total, 55 patients experienced DPPHRt and 23 DPPHRp. Of 78 patients with PNET, 55 were classified as non-functional neoplasm. The tumor diameter for this subgroup of patients is infrequently reported, but was documented in six reports to be 1.4–2.5 cm. Of 23 patients with PNET, sporadic insulinoma was observed in 16. In the subgroup of functional PNETs, malignant tumors were found in 11 patients (Table 5).

**Table 5:** Final histopathology of 89 patients with neuroendocrine tumors and 31 patients with papillary/ampullary tumors of the pancreatic head following DPPHR—tumor recurrence after follow-up of 45.8 months (mean)

	PNET					Peripapillary tumor**				
	Total pats. (benign)	Non-functional Benign/ malignant Pats.	Functional Benign/malignant Pats.	Recurrence		Papilla/ Ampulla Benign/malignant Pats.	Duodenum Benign/malignant Pats.	Peripapillary CBD Benign/malignant Pats.	Recurrence pats.	
	n/N	n	n			n	n	n	n	
DPPHRt	55	32/0	23/7*^a^		2/89^b^	7/5^c^	1/1^d^	6/6^e^		1/23^f^ 4.76 %
DPPHRp	23	23/0	0/4*			0	0	5/0^e^		

*Functional PNETs: ^a^benign: sporadic insulinoma 23 pats. malignant: gastrinoma 1 pat., carcinoid papilla 4 pats., carcinoid duodenum 1 pat., islet cell carcinoma 5 pats.;^b^gastrinoma hepatic metastases 1 pat., metastases islet-cell carcinoma 1 pat.

**Periampullary TM: ^c^benign adenoma 7 pats., T_1a_ carcinoma in adenoma of papilla 4 pats., T_1_ carcinoma of ampulla 1 pat.; ^d^benign adenoma peripapillary duodenum 1 pat., T_1_ carcinoma duodenum 1 pat., ^e^benign TMs 3 pats.; biliopancreatic maljunction 3 pats., pancreas divisum 5 pats. malignant: T_1_ carcinoma peripapillary CBD 3 pats., CBD cancer T_2_ 1 pat., biliopancreatic maljunction + CBD carcinoma T_1_ 2 pats.; ^f^metastases of advanced carcinoma of papilla/ampulla

In the subgroup of 31 patients with PATs, advanced cancer was observed in 12 patients (38.7%): most frequently T1 cancer and T1a carcinoma in adenoma of the papilla in 4 patients; T1 carcinoma of the ampulla in 1 patient; T1 of the peripapillary CBD in 3 patients; and CBD cancer T2 in 1 patient. Almost all patients with periampullary tumor experienced DPPHRt+sd (Supplemental Material, Fig. S2C) (Table 5).

### Concordance of Diagnosis of Benign Tumor

The concordance of preoperative and postoperative diagnosis of “benign tumor” based on the final histopathology was 90.57%. In patients with CNs, PNETs, or PATs, advanced malignoma was finally found in 7.05%, 12.04%, and 38.7%, respectively. A total of 18 patients who presented intraoperative signs of advanced cancer were considered for DPPHR for benign tumor and underwent intraoperative conversion to PD or re-surgery during the index hospitalization, applying a radical PD or receiving additional adjuvant chemotherapy.

### Results of Meta-analysis Comparing DPPHRt and PD for Frequency and Type of Recurrent Tumor

**Fig. 2:**
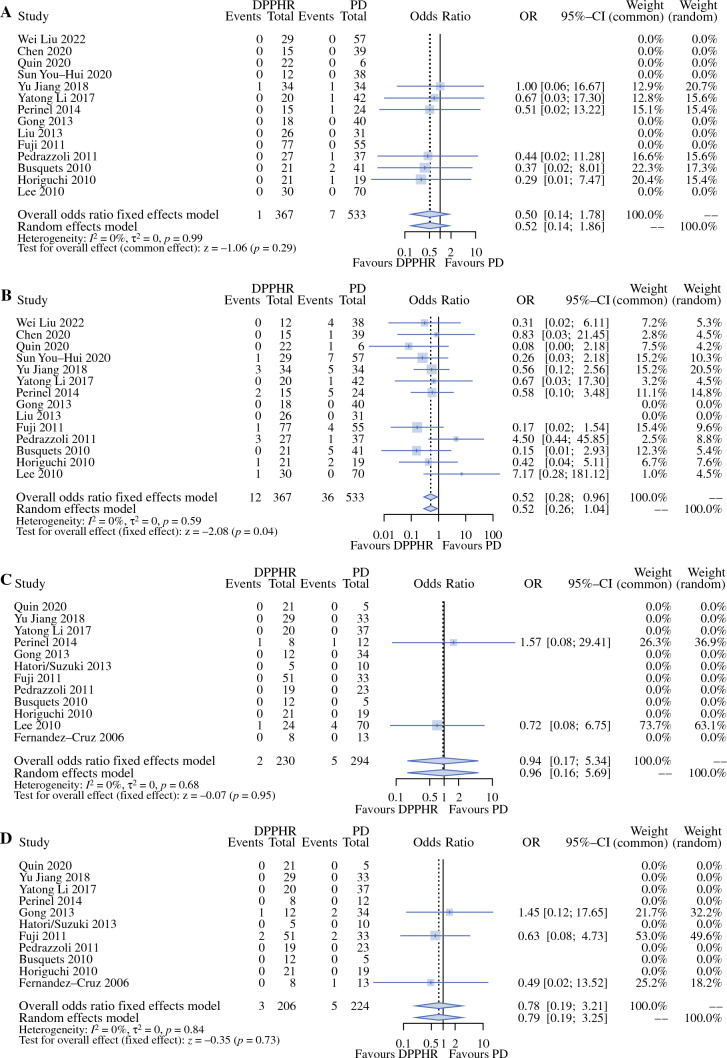
Forest plots comparing postoperative surgery-related complications and type of tumor recurrence following DPPHRt compared with PD for cystic neoplasms; **A** in-hospital mortality, **B** frequency of reoperation, **C** local recurrence at resection margin of IPMN, **D** tumor re-occurrence in remnant pancreas

**Fig. 3:**
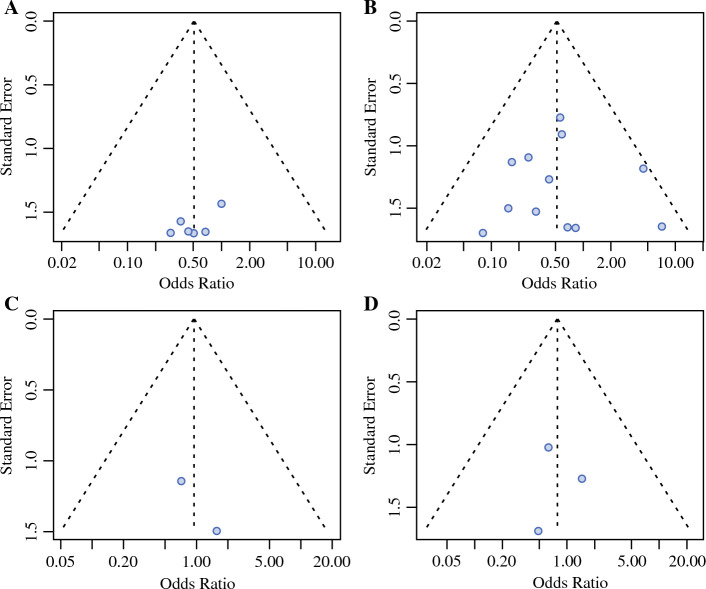
Funnel plots; **A** in-hospital mortality, **B** frequency of reoperation, **C** local recurrence at resection margin of IPMN, **D** tumor re-occurrence in remnant pancreas

The meta-analysis was based on 14 studies published between 2010 and 2022 comparing the histopathological data following DPPHRt or PD for benign tumor. Local anastomotic recurrence was observed in 2 of 230 patients following DPPHRt (0.86%) and in 5 of 294 patients following PD (1.7%) (Supplemental Material, Fig. S2C). The forest plots applying overall odds ratio fixed effects and random effects model show heterogeneity and a comparable frequency of recurrence at the resection margin after either type of resection (Supplemental Material, Fig. S2C) (*p* = 0.95). Following DPPHR, tumor reoccurrence in the remnant pancreas was observed in 3 of 206 patients (1.5%) and in 5 of 224 patients with PD (2.2%). The test for overall effect showed no difference between DPPHRt and PD (Fig. 2D) (*p* = 0.73). Regarding the type and frequency of tumor recurrence after DPPHRt in both study groups (review and meta-analysis group), 9 of 257 patients experienced tumor recurrence or reoccurrence (3.5%), respectively, in the long-term follow-up of 45.8 ± 26.6 months (Table 4). Three patients displayed IPMN recurrence at the resection margin, four patients developed reoccurrence of the tumor in the remnant pancreas, and two patients developed extrapancreatic metastases after local resection of IPMN-associated cancer. After DPPHRp, three patients developed reoccurrence of IPMN in the remnant pancreas, two of them with primarily benign IPMN (Fig. 3), one patient developed cancer of MCN at the resection margin, and one patient peritoneal metastization after intraoperative dissemination of an IPMC. (Table 4). Recurrent tumor following DPPHRt for PNET was observed in 2 of 62 patients, with one each succumbing to metastases of a gastrinoma or islet cell carcinoma (Table 5). In addition, 1 of 31 patients developed multiorgan metastases of an advanced carcinoma of the papilla/ampulla after DPPHRt (Table 5).

Comparing baseline data of the DPPHR (367 patients) and PD subgroups (533 patients) included in the meta-analysis, the following criteria displayed similar results: male/female gender and frequencies of CNs (62.7% versus 58.9%), PATs (8.4% versus 7.1%), other tumors (4.6% versus 2.8%), and cancer/malignoma (7.9% versus 7.8%). In the DPPHR group, the frequencies of PNETs (24.3 versus 13.5%; *p* < 0.001) and CP (25.9% versus 18.2%; *p* < 0.006) were higher than in the PD group. The PD group displayed a significantly higher mean age (52.5 versus 47.9 years; *p* < 0.001) and a larger tumor size (4.1 versus 3.5 cm; *p* < 0.001). In the DPPHR group, for 153 of 367 patients a duct origin of the IPMN was reported. Approximately two-thirds of patients with IPMN who underwent DPPHR displayed a BD-type, whereas in the PD group, the MD- and mixed-type IPMN prevailed in 184 of 533 patients wtih IPMN.

## Discussion

The review and meta-analysis include 729 patients who underwent duodenum-preserving pancreatic head resection for benign tumors. The analysis presents the first cumulative data of long-term outcome of 634 patients after undergoing local, parenchyma-sparing pancreatic head resection for cystic neoplasms, neuroendocrine tumors, or periampullary neoplasms.

After a mean follow-up of 45.8 months, the risk of development of a tumor recurrence after DPPHR at the resection margin was 1.21%. The reoccurrence of a benign neoplasm or an adenocarcinoma in the remnant pancreas was observed in seven patients (2.1%). The data underline that local extirpation of a benign or potentially malignant neoplasm applying DPPHR was associated with low risk of tumor recurrence. Comparing DPPHR with PD, the frequency of local tumor recurrence revealed that both surgical techniques have similarly high levels of long-term patient cure. Additionally, DPPHR has the advantages of low in-hospital mortality and a significantly lower frequency of reoperation and reinterventions compared with PD. With regard to long-term patient cure, a low risk of tumor recurrence, significantly reduced surgery-associated complications,^65^ and maintenance of metabolic pancreatic and upper-GI tract functions,^15^ DPPHR performance is to be weighed against the risk of carrying out a PD.

Predicting the risk of malignancy, international guidelines established criteria for CNs for decision-making as defined by an absolute indication for surgery, surveillance of patients for IPMN and MCN, and definition of worrisome features and high-risk stigmata.^66–69^

Diagnostic tools, including high-resolution magnetic resonance tomography (MRT), liquid biopsy, and fine needle aspiration, have led to surgeries being less frequently performed too late in the case of invasive cancer, or too early in patients with a distinct risk of malignant transformation.^70–72^

Applying parenchyma-sparing pancreatic head resection for CNs lowers the surgical risk and postoperative morbidity, even though too early or too late surgical treatment equally applies for the parenchyma-sparing surgical techniques. Most patients included in this review and meta-analysis underwent surgical treatment due to clinical symptoms.

Decision-making for surgery in asymptomatic patients under surveillance for IPMNs, MCNs, or SPNs should occur before the development of an invasive carcinoma. However, radiological factors, clinical signs, tumor markers, and even DNA analysis of the IPMN are presently not sufficiently accurate to predict the risk or the presence of malignant tumor with high sensitivity or specificity.^70–72^ Resection of suspected asymptomatic IPMN lesions through a duodenum-sparing resection is associated with low postoperative risk for complications and maintenance of metabolic and upper-GI tract functions and is thereby the basis for a cancer-preventive surgical treatment.^72,73^

In a recently published analysis of a large IPMN cohort of 1074 patients including benign and advanced neoplasms, recurrence rate was 14.4% at a median of 24 months.^74^ Recurrence was defined as radiographic or histologic diagnosis of a metachronous tumor in the remnant pancreas or appearance of a tumor outside the pancreas.^74^ Recurrence in this analysis included tumor growth at the resection margin, metachronous tumor reoccurrence in the remnant pancreas, or extrapancreatic, metastatic tumor growth in the follow-up time. The recurrence rates following DPPHR among 258 patients with benign, noninvasive IPMN, including the 24 patients with cancer arising in association with IPMN, was 5.03% after a mean follow-up of 45.8 months. Four patients developed tumor recurrence located at the resection margin tissue, six patients experienced reoccurrence of an IPMN or a PDAC in the remnant pancreas, and three patients developed extrapancreatic metastization. Frozen section control of the resection margin is recommended in any case of local tumor extirpation. For cystic neoplasms of the pancreatic head, tumor size or tumor location close to the duodenal wall and/or compression of the intrapancreatic CBD are criteria for selection of the type of DPPHR. DPPHRt (type II) was used for BD- and mixed-type IPMN and for MCN. DPPHRt+sd (type III) including segment resection of the peripapillary duodenum and the intrapancreatic CBD was applied for MD-IPMN of the pancreatic head. DPPHRp for SCN and SPN was associated with the advantage of preserving the intrapancreatic CBD and minimizing tissue loss of the pancreatic head. Performing total or partial DPPHR for SPN is considered a major advantage and as a cancer-preventive treatment, because most patients at the child and adolescent age are female.^34,55^

During the long-term outcome, seven patients developed metachronous tumor appearance in the remnant pancreas, clearly as a consequence of a multifocal disease. This may be attributed to the difference in the fundamental biology of the disease driving the process of malignant transformation.^75,76^ These data underline that a postoperative surveillance protocol should be maintained after local, parenchyma-sparing resection of the pancreatic head for patients with the risk for multifocality of the neoplasm. The development of extrapancreatic cancer recurrence after surgical treatment of IPMN was equally low following PD and DPPHR. Interestingly, 9 of 25 patients, who were classified in the final histopathology as micro-carcinoma or focal cancer of IPMN, did not develop local or systemic recurrence in the mean follow-up time of 45 months.

Habib et al. found that the pattern and timing of recurrence after resection of an invasive carcinoma arising in association with IPMN was significantly longer for recurrence at resection margin (21.6 months) than for systemic recurrence (11.4 months).^26^ Independent predictors of systemic recurrence are R1 margin positivity for high-grade dysplasia (HGD) or invasive cancer and poor differentiation. Regarding the histologic pattern of IPMNs, data from Koh et al. demonstrated that the pancreatobiliary subtype was associated with a higher risk for recurrence, whereas the gastric subtype was found to have a lower risk for recurrence.^77^ Systemic cancer recurrence was observed after DPPHR of advanced cancer originating from cystic neoplasm in three patients. Distant metastization after resection of cystic neoplasm predominantly develop in up to three postoperative years, which was covered by the follow-up time.

In this review, 21.1% of patients with IPMN, whose pattern of dysplasia of the neoplasm was reported, exhibited severe dysplasia in the final histopathology. None of the patients with severe dysplasia of IPMN were reported to have developed tumor recurrence after DPPHR. Local, parenchyma-sparing pancreatic head resection using DPPHRt (types II or III) for benign IPMN containing severe dysplasia is a safe treatment to cure the patient. On the basis of genomic analysis, data from Noe et al. revealed an average window of more than 3 years between HGD and the development of an invasive pancreatic cancer.^78^ IPMN with the presence of HGD is considered to be a risk factor for the subsequent development of pancreatic ductal adenocarcinoma.^79–82^ Therefore, presence of HGD in IPMN is a criterion for cancellation of surveillance and a change without delay to surgery, applying an adequate parenchyma-sparing surgical treatment.

### PNETs

DPPHR was performed in 89 patients with PNETs. The final histopathologic investigation revealed predominantly non-functional neoplasms. Tumor size was 2.97 and 3.7 cm (*p* ≤ 0.001) after partial or total DPPHR, respectively. Current 2016 guidelines of the European Neuroendocrine Tumor Society (ENET) recommend resection for all functional, sporadic PNETs regardless of tumor size.^83^ For non-functional PNETs, surveillance is favored for tumors ≤ 2 cm, except for patients with symptoms or higher tumor grade or presence of enlarged lymph nodes.^84^ For non-functional PNETs of the pancreatic head, a local DPPHRp was preferentially applied and displayed an in-hospital mortality of 0%. For PNETs ≥ 3 cm in size or the presence of sporadic insulinoma, duodenum- and CBD-preserving total head resection was applied. Lymph node allocation is recommended for each patient when undergoing local or classical resection for PNET.

DPPHRt with segment resection of the peripapillary duodenum and intrapancreatic CBD was carried out in five patients with carcinoid tumors, one of the duodenum and four of the papilla. ENET guidelines recommend surveillance for non-functional PNETs ≤ 2 cm if the tumor is grade 1 or low grade 2 and asymptomatic.^83^ However, recently published results presented data to expand the scope of surgical removal of tumors with a size of 1–2 cm.^85–87^ The increase in the recognition of PNETs has led clinicians to reconsider operative approaches to small, non-functional tumors of 1–2 cm.^88^ Tumor enucleation is the most favored surgical treatment for small, non-functional PNETs.^86,88^ However, tumor enucleation for PNETs located in the pancreatic head have a high risk of pancreatic fistula B or C, following opening of one of the pancreatic main ducts. Injuring of the pancreatic main ducts is frequently associated with local complications, increasing risk for re-interventions, and leading to longer hospital stays.^89,90^ A survival benefit was found for resection of PNETs > 1 to < 2 cm out of 3243 cases of < 2 cm when performing a classical pancreatic head resection or tumor enucleation.^88^ DPPHR for pancreatic head PNETs including sporadic non-functional and functional PNETs was performed in 89 patients with an in-hospital mortality of 0%. DPPHR for PNETs of the pancreatic head was associated with low levels of POPF B+C, low frequency of re-interventions, and maintenance of endocrine and exocrine pancreatic functions. Histopathologic diagnosis and staging of neuroendocrine tumors of the pancreatic head should also be based on lymph node investigation. In the group of partial DPPHR, lymph node dissection for PNETs was not routinely performed; the staging of the patients with non-functional PNET after partial DPPHR was incomplete.

### Periampullary Tumors

The concordance between pre- and postoperative diagnosis of benign periampullary tumor was 61.3%. In the final histopathology, 12 of 31 patients exhibited T1 cancer in association with villous or tubulovillous adenoma. Villous or tubulovillous adenomas are the most common benign lesion, bearing a significant malignant potential.^91^ In this series of 31 periampullary tumors, 38.7% displayed advanced cancer in the final histopathologic examination, most of them T1 stage cancer. All patients were preoperatively managed by endoscopic surveillance programs including endoscopic resection procedures. The patients were finally referred for surgical treatment. Histopathological assessment of biopsy material revealed carcinoma in small adenomas of the papilla in more than 20% and in large villous adenomas in up to 60%.^92,93^ Except for 5 patients, who exhibited a benign peripapillary common bile duct lesion, 26 patients had a DPPHRt with segment resection of the peripapillary tumor-bearing segment of the duodenum and resection of the CBD. Of note, final histopathology in five additional patients revealed carcinoid tumor of the papilla (four patients) or the peripapillary duodenum (one patient).These patients are listed in the group of PNETs according to international guidelines (Table 5).The in-hospital mortality was 0% for 31 patients with PAT. DPPHRt with segment resection of the peripapillary duodenum and CBD is recommended for peripapillary and ampullary adenomas, which have histologically severe dysplasia or are associated with T1 carcinoma. Dissection of N1 and N 2 lymph nodes around the pancreatic head was performed in association with DPPHR for tumor staging without extension of the surgical procedure. Local tumor recurrence after DPPHRt+sd was not observed in the long-term follow-up, except for one patient who succumbed to liver metastization following resection of advanced cancer of the papilla. Applying total DPPHR for patients with advanced papillary/ampullary cancer, notably for patients with distal CBD cancer, is an inadequate surgical treatment. Whipple resection is recommended.

## Summary

DPPHR was performed in 729 patients for benign tumors of the pancreatic head, premalignant cystic neoplasms, neuroendocrine tumors, or low malignant peripapillary/ampullary adenomas. The concordance of the preoperative diagnosis of benign tumor with the final histopathology was 90.57%. In-hospital mortality was 0.26% after DPPHR and 1.31% after PD. After a follow-up of a mean of 45.8 months, 4.11% of the patients with IPMN/MCN developed recurrent tumor, of whom only four succumbed to recurrent tumor at the resection margin. Of 89 patients with PNETs, 2 patients (2.3%) experienced a metastatic recurrence. In-hospital mortality after DPPHR for PNETs was 0%. For patients suffering from premalignant CNs, benign PNETs, or low malignant PATs, DPPHR ensures long-term cure and is associated with a significantly lower risk for surgery-associated complications and low in-hospital mortality when compared with PD.

### Limitations

This systemic review and meta-analysis has several limitations. Generally, the inclusion of cohort studies based on a small number of patients increases the risk of bias and limits the conclusions. A total of 5 of the 14 studies used for meta-analysis were retrospective, controlled cohort studies. The comparison of the results of DPPHRt and PD was published, with one exception, in the past 11 years. However, the data from studies of the review group span a reporting period of 27 years. The inclusion of non-comparative studies is of limited evidence.

The incomplete reporting of standard criteria for histopathologic differentiation, and of metrics for postoperative outcome in both groups (DPPHR and PD), limits the conclusions. The results of randomized controlled trials are warranted to establish high-quality clinical evidence regarding the advantages and limitations of DPPHRt compared with PD and the use of DPPHR compared with tumor enucleation.

### Supplementary Information

Below is the link to the electronic supplementary material.

Supplementary file1 Fig. S2 Supplementary file: (DPPHR type I/II/III); (A) type I: partial pancreatic head resection (DPPHRp) - reconstruction with side-to-side pancreaticojejunostomosis, (B) type II: total DPPHR (DPPHRt) - with preservation of the duodenum and the intrapancreatic common bile duct; reconstruction using the first jejunal loop, (C) type III: reconstruction after DPPHRt+sd with segment resection of the peripapillary duodenum and resection of the intrapancreatic common bile duct (PDF 181 kb)
