# Supplementary material for: Long-Term Oncologic Outcome following Duodenum-Preserving Pancreatic Head Resection for Benign Tumors, Cystic Neoplasms, and Neuroendocrine Tumors: Systematic Review and Meta-analysis
Source: Ann Surg Oncol. 2024 Apr 5;31(7):4637–53. doi: 10.1245/s10434-024-15222-y (PMC11164799; doi:10.1245/s10434-024-15222-y)

Parenchyma-sparing pancreatic head resection - Beger et al.

Electronic Supplemental Material: Figures S2A - S2C

Fig. S2A: Type I - Partial Pancreatic Head Resection (DPPHRp) - Reconstruction with side-to-side Pancreaticojejunostomosis (similar to Berne modification)

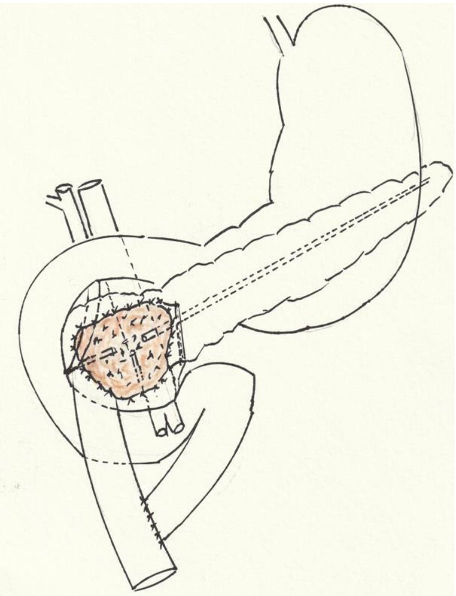

Fig. S2B: Type II - Total DPPHR (DPPHRt) - with Preservation of the Duodenum and the Intrapancreatic Common Bile Duct; Reconstruction Using the First Jejunal Loop

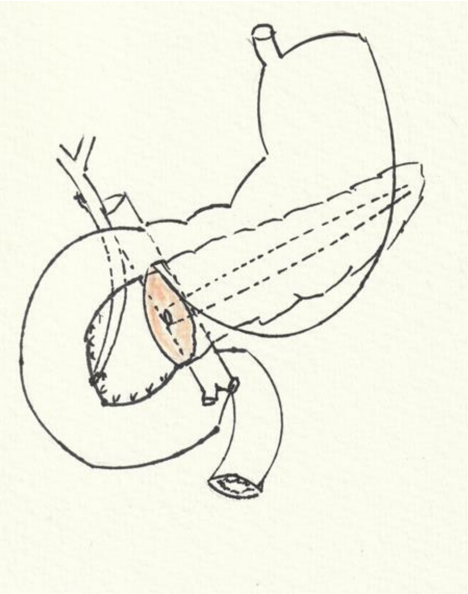

Fig. S2C: Type III - Reconstruction after DPPHRt + sd with Segment Resection of the Peripapillary Duodenum and Resection of the Intrapancreatic Common Bile Duct

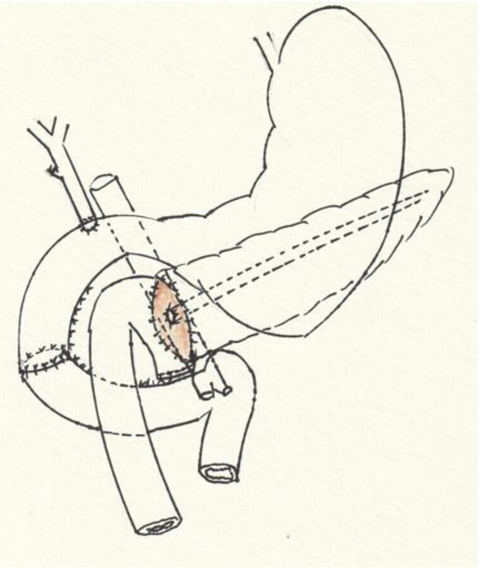

Supplement: Supplementary file 1 — Supplementary file1 Fig. S2 Supplementary file: (DPPHR type I/II/III); (A) type I: partial pancreatic head resection (DPPHRp) - reconstruction with side-to-side pancreaticojejunostomosis, (B) type II: total DPPHR (DPPHRt) - with preservation of the duodenum and the intrapancreatic common bile duct; reconstruction using the first jejunal loop, (C) type III: reconstruction after DPPHRt+sd with segment resection of the peripapillary duodenum and resection of the intrapancreatic common bile duct (PDF 181 kb) [file 10434_2024_15222_MOESM1_ESM.pdf]
